# Pituitary apoplexy can mimic acute meningoencephalitis or subarachnoid haemorrhage

**DOI:** 10.1186/1865-1380-4-63

**Published:** 2011-10-05

**Authors:** Ahmed-Ramadan Sadek, Stephen Gregory, Thiagarajan Jaiganesh

**Affiliations:** 1 Wessex Neurological Centre, Southampton University Hospitals NHS Trust, Tremona Road, Southampton SO16 6YD, UK; 2 Division of Clinical Neurosciences, School of Medicine, University of Southampton, Tremona Road, Southampton SO16 6YD, UK; 3 Radiology Department, St. Georges Hospital, Blackshaw Road, Tooting, London, SW17 0QT, UK; 4 Emergency Department, St. Georges Hospital Blackshaw Road, Tooting, London, SW17 0QT, UK

## Abstract

Pituitary apoplexy is an uncommon but life-threatening condition that is often overlooked and underdiagnosed. We report a 45-year-old man who presented to our emergency department with a sudden onset headache, acute confusion, signs of meningeal irritation and ophthalmoplegia. An initial diagnosis of acute meningoencephalitis was made, which was amended to pituitary apoplexy following thorough investigation within the emergency department.

A 45-year-old man was brought to our emergency department by ambulance with a history of sudden onset of frontal headache and acute confusion. His wife provided the history. There was no significant past medical history of diabetes, hypertension, recent travel abroad, exposure to sick contacts, involvement in outdoor pursuits such as hiking/cave diving, or trauma. He worked in a bank and had been well until 24 h prior to the onset of sudden headache, which was gradually worsening in nature and associated with increasing confusion. The patient's wife reported that he had neither experienced any fevers, night sweats, or coryzal symptoms nor received any recent vaccinations. He was not on any regular medications. He was a non-smoker and occasionally consumed alcohol. There was no significant family history. On examination in the ED, his temperature was 37.6°C, his pulse was 110/min, and he was normotensive and normoglycaemic. A macular blanching rash was noted over the patient's trunk. The patient was disoriented to time and place. Neurological examination revealed reduced GCS (11/15-E3, M6, V2), marked neck stiffness, a positive Kernig's sign and a right sixth nerve palsy.

A provisional diagnosis of acute meningoencephalitis was made and the patient was started on a course of intravenous antibiotics with benzyl penicillin 1.2 g, cefotaxime 2 g and acyclovir 750 mg. Baseline blood investigations revealed hyponatraemia (122 mmol/l), a white-cell count of 11 × 10^9^/l and a C-reactive protein > 250. Due to the sudden onset of the symptoms and lack of prodrome, an urgent CT head scan was performed to rule out a cerebrovascular event. The scan demonstrated an enlarged pituitary gland (3 cm in diameter) with impingement of the optic chiasm. The centre of the enlarged pituitary gland was noted to be hypodense in comparison to its periphery, which was consistent with a diagnosis of pituitary apoplexy. A subsequent MRI confirmed the diagnosis (Figure 1) of an enlarged sella containing abnormal soft tissue with increased signal intensity suggestive of haemorrhage (Figure 1A).

Post-MRI a lumbar puncture was performed revealing glucose 3.4 mmol/l, protein 1.0 g/l, red cells of 53/mm^3 ^and white cells of 174/mm^3 ^with predominant neutrophilia. No organisms were seen, and CSF cultures and HSV DNA tests were found to be negative. Endocrinological investigations demonstrated low concentrations of thyroid hormones [TSH: 0.14 mIu/l (0.35-5.5 mlU/l), FT3: 1.1 nmol/l (1.2-3.0 nmol/l), FT_4_: 9.6 pmol/l (8-22 pmol/l)], gonadal hormones (LH: < 1 u/l) and prolactin: 16 u/l (<450 u/l). Serum FSH was 2.9 u/l (0.8-11.5 u/L) and cortisol 575 nmol/l (450-700 nmol/l). The patient was treated for hypopituitarism based on clinical and radiological findings with intravenous fluids, hydrocortisone (100 mg) and thyroxine (50 μg) as loading doses in the ED.

Within 24 h of commencement of therapy the patient's GCS rose to 15, and within 48 h there was marked improvement in the right sixth cranial nerve palsy. Formal visual field assessment demonstrated temporal visual field loss in the left eye. The patient was discharged to his usual residence a week later and follow-up was organised with both the endocrinologists and ophthalmologists. Follow-up MRI demonstrated that there was no significant change in either size or signal characteristics of the pituitary fossa mass (Figure 1B).

## Discussion

Pituitary apoplexy was first described in 1898 by Bailey [1]. It is characterised by headache, visual loss, ophthalmoplegia and altered mental status as a result of a sudden infarction or haemorrhage within the pituitary gland. The peak incidence is around the 5th - 6th decades of life with a male to female ratio of 2 to 1. The diagnosis is frequently complicated by signs and symptoms that resemble those of other intracranial pathological entities such as meningoencephalitis [2], meningitis [3] or subarachnoid haemorrhage.

**Figure 1:**
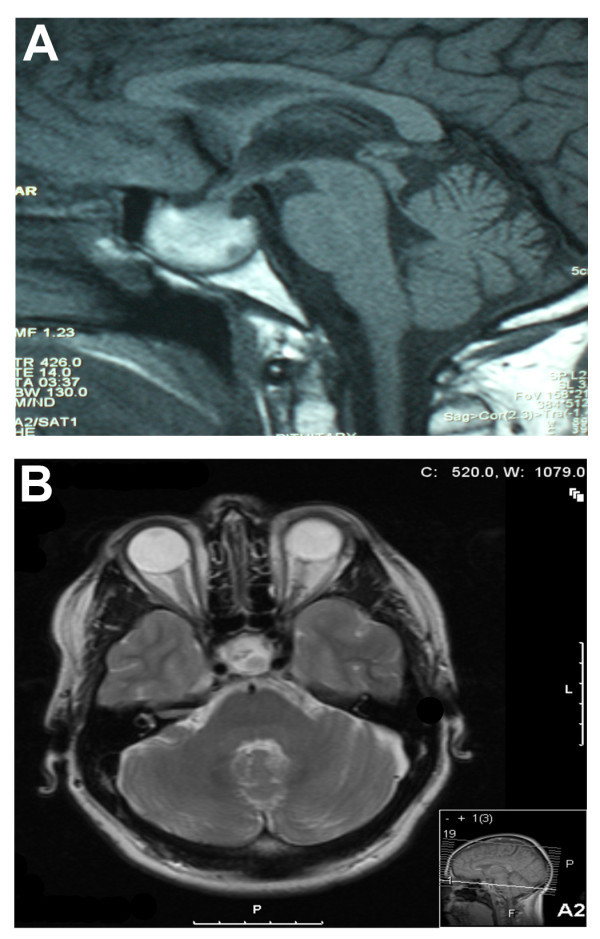
**MR imaging of pituitary apoplexy**. **a **A sagittal T1 image of the pituitary gland. The enlarged sella containing an abnormal soft tissue with increased signal intensity is suggestive of haemorrhage. **b **A transverse T1 image of the pituitary gland 2 months following presentation. The sella is expanded and contains abnormal soft tissue that is of low T1 signal. A small area of high T1 signal in the superior aspect of this is taken to represent normal posterior pituitary signal. There are no features of haemorrhage on this later scan.

Headache is the most commonly (95-100%) reported symptom [4], and is sudden and severe in nature with a tendency to be bifrontal or retro-orbital. It is thought to be as a result of stretching and irritation of the dura mater overlying the sella turcia that is supplied by the 5th cranial nerve. The release of necrotic and haemorrhagic material into the suprasellar subarachnoid space [3] either through the diaphragmatic aperture or through rupture of the sellar diaphragm [5] gives rise to symptoms consistent with meningoencephalitis and pleocystosis. Both blood in the subarachnoid space and/or adrenal insufficiency can present as fever, with the former heralding symptoms of meningeal irritation, seizures and more rarely paresis secondary to vasospasm (or compression of the carotid artery against the anterior clinoid process). Compression of the optic nerve and chiasm as a result of pituitary apoplexy can give rise to visual field defects such as bitemporal haemianopia. There is evidence to suggest that the third followed by the sixth and rarely the fourth cranial nerves can be affected as a result of lateral expansion of pituitary haemorrhage and necrosis [4]. There may also be a Horner's sign secondary to involvement of the sympathetic plexus.

Our patient was noted to be hyponatraemic; this is most likely to be dilutional secondary to inappropriate secretion of anti-diuretic hormone. Hypernatremia may also be present as a result of cranial diabetes insipidus. Endocrinological abnormalities in pituitary apoplexy are due to hormone deficiency as a result of destruction of both the hypophyseal and hypothalamic tissue. This may be as a result of invasion of a primary tumour. Pituitary apoplexy is associated with all types of adenomas. However, the non-functional tumours, such as null cell adenomas and oncocytomas, predominate, as in our case. The apoplectic episode is often the initial presentation of most cases of undiagnosed pituitary tumours. In all cases, MRI is recognised as the gold standard investigation as CT scan can identify fewer than 50% of all cases of pituitary haemorrhage [6].

The precipitants of pituitary apoplexy can be categorised into four main categories such as: reduced cerebral blood flow (i.e. hypoperfusion states), increased intracranial pressure (i.e. coughing, sneezing and space-occupying lesion) leading to infarction, stimulation of the pituitary gland with resultant increased blood flow (i.e. exogenous oestrogens, pregnancy, bromocriptine and gonadotrophin-releasing hormone analogues) and anticoagulation resulting in haemorrhage within the gland [7]. Other predisposing factors include head trauma, diabetes and radiotherapy [8]. Altered mental status may be more frequent in patients with associated diseases [7]. In most cases pituitary apoplexy is unpredictable, as most patients do not have any identifiable triggering event.

## Conclusions

Pituitary apoplexy, although variable in its clinical appearance, should be considered as a part of the differential diagnosis in any patient who presents to the emergency department with an abrupt neuro-ophthalmological deterioration associated with headache. Early diagnosis is essential as prompt treatment improves survival and reduces the risk of permanent visual impairment.

## Consent

Written informed consent was obtained from the patient for publication of this case report and any accompanying images. A copy of the written consent is available for review by the Editor-in-Chief of this journal.

## Abbreviations

GCS: Glasgow Coma Scale; CT: Computerised tomography; MRI: Magnetic resonance imaging.

## Competing interests

The author declares that they have no competing interests.

## Authors' contributions

ARS wrote the first draft of the paper and coordinated the review of all the drafts. SG reviewed all radiographic images and reviewed all drafts. JT reviewed and commented on all the drafts of the paper and on all radiographic images.
